# Nb and Ni Nanoparticles Anchored on N-Doped Carbon Nanofiber Membrane as Self-Supporting Anode for High-Rate Lithium-Ion Batteries

**DOI:** 10.3390/nano12213724

**Published:** 2022-10-23

**Authors:** Yezheng Zhang, Shan Zhang, Shuo Zhao, Yingxue Cui, Jiabiao Lian, Guochun Li

**Affiliations:** Institute for Energy Research, Jiangsu University, Zhenjiang 212013, China

**Keywords:** lithium-ion batteries, flexible anode, electrospinning, nanofiber membrane, self-supporting

## Abstract

A flexible N-doped carbon nanofiber membrane loaded with Nb and Ni nanoparticles (Nb/Ni@NC) was prepared using electrospinning technology and a subsequent thermal annealing method and used as a self-supporting anode material for lithium-ion batteries. The Nb/Ni@NC nanofiber membrane had excellent flexibility and could be folded and bent at will without fragmentation and wrinkling; the nanofibers also had a uniform and controllable morphology with a diameter of 300–400 nm. The electrochemical results showed that the flexible Nb/Ni@NC electrode could deliver a high discharge capacity of 378.7 mAh g^−1^ after 200 cycles at 0.2 A g^−1^ and an initial coulombic efficiency of 67.7%, which was higher than that of the pure flexible NC anode in contrast. Moreover, a reversible discharge capacity of 203.6 mAh g^−1^ after 480 cycles at 1.0 A g^−1^ was achieved by the flexible Nb/Ni@NC electrode with a capacity decay for each cycle of only 0.075%, which showed an excellent rate capability and cycling stability.

## 1. Introduction

With the development of economies and societies, advanced energy storage technology is playing an increasingly important role in modern life. In the context of increasing demand for various mobile devices and electric vehicles, an excellent energy storage device is essential. As the lightest metal in the periodic table, lithium is ideal for making powerful portable batteries. In the era of rapid development of lithium-ion batteries, the development of more efficient and larger capacity batteries is an irresistible trend [1,2,3]. However, current batteries are still thick, heavy, hard, and not easy to carry. Therefore, the innovation of cell technology needs to develop in the direction of portable, thin, high-capacity, and flexible devices such as folding displays, touch screens, implantable medical devices, and wearable sensors, all of which promote the development of flexible electronic products [4,5,6,7,8,9]. According to the market development demand, the market growth of flexible electronic products will be rapid and lasting, not only in the field of electronic equipment, but also in various fields in which it will be applied in the future. In recent years, an increasing number of researchers have studied flexible LIBs, even whether they can be bent and folded [10], but it is still a challenge to find materials with an excellent electrochemical performance. Seeking a suitable flexible LIB requires the development of flexible components such as flexible electrodes, partitions, and electrolytes [11,12,13,14,15,16,17]; such flexible materials require: (I) a high conductivity to achieve the rapid transfer of electrons; (II) a high flexible strength and stable deformation; and (III) the desired electrochemical performance.

The fiber electrode, which occupies a position that is indisputable, has an excellent flexibility, but compared with the general rigid or flat cells, the performance of fiber cells is relatively low [18,19]. Various nanomaterials and fiber materials have been explored to achieve suitable fiber/cable-type cells, including flexible carbon fiber sheets (CFC) [20], graphene and graphene-modified materials [21], carbon nanotubes (CNTs) [22], Mxene 2D material [23], polymers and other composite materials, etc. [24,25,26,27]. Due to its unique flexible fiber structure, carbon nanotube fiber has been applied in flexible LIB anodes [28,29,30,31]. However, simple carbon nanotubes or graphite fibers still have other problems such as insufficient reaction sites, poor electrical conductivity, and a low theoretical capacity (372 mAh g^−1^), so modification or doping of carbon materials is a useful method. Some transition metal atoms can enhance the conductivity and catalytic performance of the material. Materials such as platinum nanoparticles, silver nanowires, and graphene sheets can also be introduced as functional components to improve the conductive properties of the materials [32,33,34,35]. Another way to improve conductivity is by doping heteroatoms such as those of nitrogen, sulfur and boron [36,37,38,39]; these atoms can be doped uniformly on carbon nanofibers (CNFs), which not only improves the electrical conductivity but also facilitates charge transport. Wang et al. proved through experiments and the density functional theory that high conductivity, fast lithium ion kinetics, fast interfacial charge transfer, and strong physical/chemical adsorption can be achieved by doping nitrogen and highly dispersed cobalt catalysts [40].

It is worth mentioning that it was found that carbon-doped Nb TiO_2_ nanofibers showed a higher electron conductivity (0.12 vs. 5 × 10^−6^ S cm^−1^) and surface area (86 vs. 17 m g^−1^) than those without carbon doping [41]. Chen et al. proved that nickel nanoparticles could graphitize amorphous carbon into graphite carbon and that the use of graphene/cobalt-based nitrogen doping also produced external defects in graphite carbon materials (graphene and carbon nanotubes), so the materials obtained showed a better electrochemical performance [42,43,44]. A carbon-supported multimetal monoatomic catalyst had a synergistic effect similar to that of the metal alloy catalyst. The nanoparticle pair could be used as a double catalytic site that was conducive to regulating the activity and selectivity of the product [45].

In this work, we designed a flexible Nb/Ni@NC nanofiber electrode as a self-supporting anode material for lithium-ion batteries. The synergy of the Nb, Ni, and N doping promoted LIBs transport and the carbon nanofiber with stacked features for the nucleation of Li^+^ provided ample nucleation sites. At the same time, the larger gap between the nanorods could alleviate the volume expansion of the LIBs well and enhanced the safety and stability of the cell [46]. The electrochemical test results also confirmed that the material had a good electrochemical stability.

## 2. Materials and Methods

### 2.1. Material Synthesis

The N-doped carbon nanofiber membrane loaded with Nb and Ni nanoparticles (denoted as Nb/Ni@NC) was prepared using electrospinning technology plus a thermal annealing process. Firstly, 2 mmol of C_10_H_25_NbO_5_ and 1 mmol of Ni(CH_3_COO)_2_ + 4H_2_O (Nb:Ni molar ratio of 2:1) were added into 4 mL of N, N-dimethylformamide (DMF) and stirred vigorously for 6~8 h; meanwhile, 1 g of polyacrylonitrile (PAN, MW = 150,000) was dissolved in 6 mL of DMF and stirred vigorously for 6~8 h. Secondly, the two solutions were mixed and stirred for another 12 h to obtain a uniform green suspension. Thirdly, the green mixture was then inhaled into a 10 mL syringe for electrospinning. Fourthly, the collected sample film was preoxidized at 250 °C and then carbonized at 700 °C under a N_2_ atmosphere for 2 h to obtain the flexible Nb/Ni@NC nanofiber membrane. The synthesis method for NC was the same except that C_10_H_25_NbO_5_ and Ni(CH_3_COO)_2_ + 4H_2_O were not added. Finally, the flexible Nb/Ni@NC and NC nanofiber membranes were punched into a wafer-shaped electrode with diameter of 12 mm (Figure S1).

### 2.2. Characterization

The X-ray diffraction (XRD) measurement was performed with an X-ray diffraction instrument (German Bruker D8 with Cu Kα, λ = 1.54056A). The field-emission scanning electron microscopy (FESEM) was conducted on a JSM-7800F instrument. The transmission electron microscopy (TEM) was conducted on a JEM 2100F instrument and included energy-dispersive spectroscopy (EDS) to observe the morphology and elemental distribution of the samples. X-ray photoelectron spectroscopy (XPS) was performed using a Thermo Scientific K-alpha ray photoelectron spectrometer.

### 2.3. Electrochemical Measurements

The punched wafers of the flexible Nb/Ni@NC and NC nanofiber membranes were used directly as self-supporting working electrodes to assemble Li half-cells without adding commonly used conductive additives and binders; the mass loading of the active material was calculated to be around 1–1.4 mg cm^−2^. Lithium foil was used as the counter electrode and reference electrode, the separator was a commercial Celgard PP separator, and the electrolyte was 1.0 M of lithium hexafluorophosphate (LiPF6) dissolved in ethylene carbonate (EC) and diethyl carbonate (DEC) (*v*:*v* = 1:1) with 5.0 vol % of 4-fluoro-1,3-dioxolan-2-one (FEC) as an electrolyte additive. The assembly of 2032-type coin cells was carried out in a glove box filled with argon gas (H_2_O < 0.1 ppm, O_2_ < 0.1 ppm). A LAND CT2001A tester was used for galvanostatic charge/discharge measurements within a voltage range of 0.01–3 V. Cyclic voltammetry and electrochemical impedance spectroscopy (EIS) tests were carried out on an electrochemical workstation (Gamry). The scan rate for CV measurements was 0.1 mV s^−1^, the frequency range of EIS was 10 mHz to 100 kHz, and the amplitude was 10 mV.

## 3. Results and Discussion

**Figure 1:**
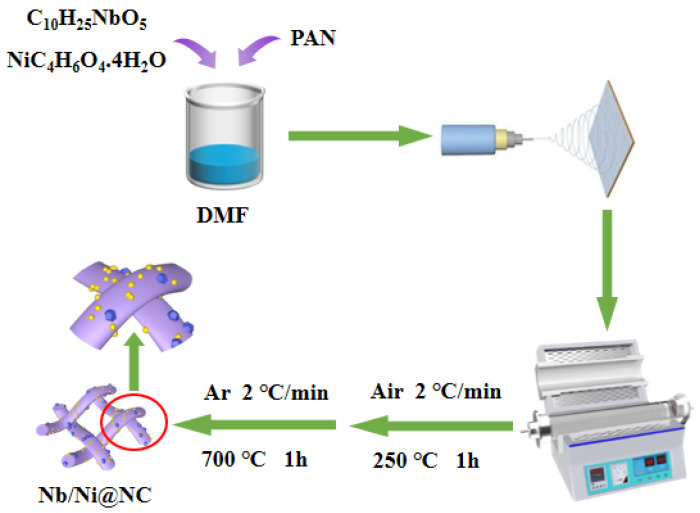
Schematic diagram for the preparation of flexible Nb/Ni@NC nanofiber membrane.

Figure 1 presents a detailed schematic diagram for the preparation of the flexible Nb/Ni@NC nanofiber membrane, which was firstly prepared using electrospinning technology to obtain the precursor and then using thermal annealing to obtain the target product. The as-prepared Nb/Ni@NC nanofiber membrane had an excellent flexibility that could be quickly restored to normal after being folded and bent without fragmentation and wrinkling, facilitating the assembly of flexible energy storage devices (Figure S2a–c). In contrast, the flexible NC nanofiber membrane had a poor toughness and was prone to fracturing after being folded (Figure S2d–f). These results indicated that the introduction of metal nanoparticles could increase the flexibility of the membrane material and reduce its brittleness.

**Figure 2:**
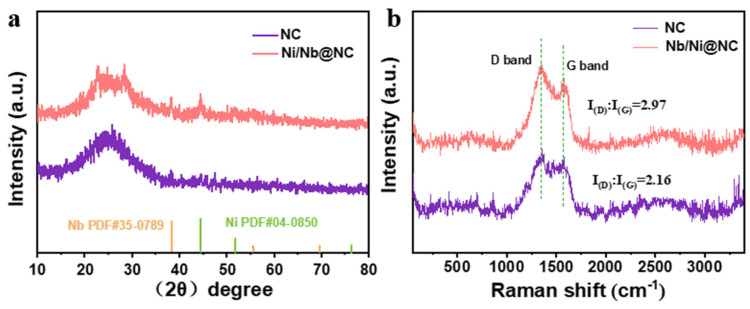
(**a**) XRD pattern of Nb/Ni@NC and NC nanofibers; (**b**) Raman spectra of Nb/Ni@NC and NC nanofibers.

Figure 2a shows the X-ray diffraction (XRD) patterns of the Nb/Ni@NC and NC nanofiber membranes. It can be seen that there were several more diffraction peaks for Nb/Ni@NC than for NC that corresponded to the characteristic peaks of Nb (JCPDS No. 35-0789) and Ni (JCPDS No. 04-0850). In addition, both Nb/Ni@NC and NC had an amorphous carbon peak at about 25°, suggesting that relatively pure Nb/Ni@NC nanofibers were successfully synthesized. The Nb/Ni@NC and NC samples were further characterized using Raman spectroscopy (Figure 2b). Two characteristic peaks of carbon crystal were detected: a D band near 1350 cm^−1^ and a G band near 1580 cm^−1^. The I(D)/I(G) value for the Nb/Ni@NC nanofiber (2.97) was larger than that of the NC nanofiber (2.16), indicating that there were more crystal defects of carbon atoms in the Nb/Ni@NC nanofiber than those in the NC nanofiber. Therefore, the number of defects caused by the introduction of Nb and Ni metal elements was significantly greater than those caused by the mere addition of N atoms.

**Figure 3:**
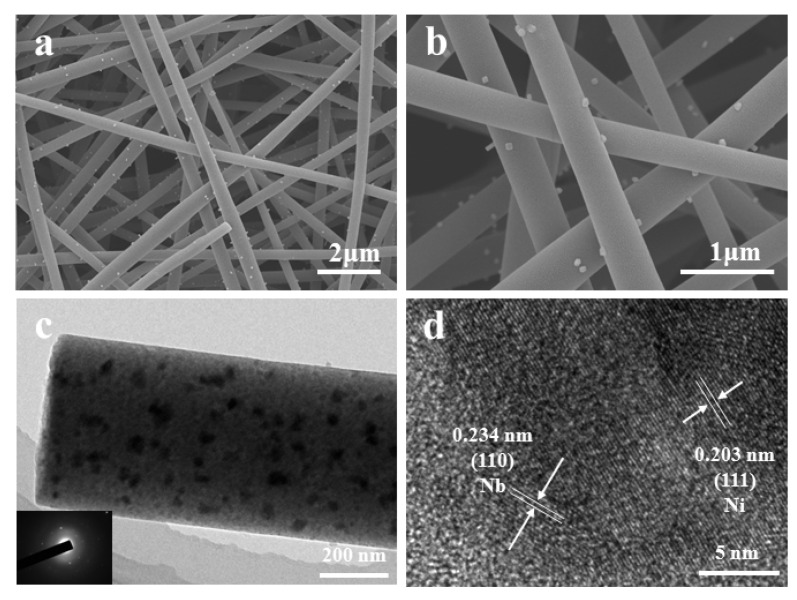
(**a**,**b**) SEM images of Nb/Ni@NC nanofibers with low/high magnification; (**c**,**d**) low-magnification TEM image and high-resolution TEM image of Nb/Ni@NC nanofiber.

Figure 3 shows the microstructure of the Nb/Ni@NC nanofibers characterized using scanning electron microscopy (SEM) and transmission electron microscopy (TEM) with low/high magnification. As can be seen, the morphology of the nanofibers was uniform and the diameter was about 300–400 nm (Figure 3a,b). These nanofibers were interwoven without dense stacks and there were plenty of spaces between them to withstand the expansion and contraction during electrode operation. In addition, it can be observed that there were quite a few nanoparticles that were distributed on the surface of the Nb/Ni@NC nanofibers while the surface of the NC nanofibers was smooth based on the SEM observation (Figure S3a,b). Thus, the morphology of the Nb/Ni@NC nanofibers was further characterized using transmission electron microscopy (TEM). As shown in Figure 3c, many nanoparticles that were of variable sizes were indeed distributed on the surface of the nanofibers. The high-magnification TEM image in Figure 3d further proved that two types of lattice fringes were distinguished in the sample: the lattice spacing of 0.234 nm was ascribed to the lattice plane of Nb (110) while the lattice spacing of 0.203 nm corresponded to the lattice plane of Ni (111), which was consistent with the XRD pattern data. In addition, the bright small particles scattered on the surface of the nanofibers found in the SEM observation were further verified as metal Ni nanoparticles using scanning transmission electron microscopy technology (STEM, Figure S4); the EDS mappings further showed that there were four elements, namely C, N, Nb, and Ni. The sizes of the metal Ni and metal Nb nanoparticles were about 17–32 nm and 3–5 nm, respectively (Figure S6). Obviously, these elements were evenly distributed on the nanofibers without stacking phenomenon, indicating the successful doping of Nb, Ni, and N.

**Figure 4:**
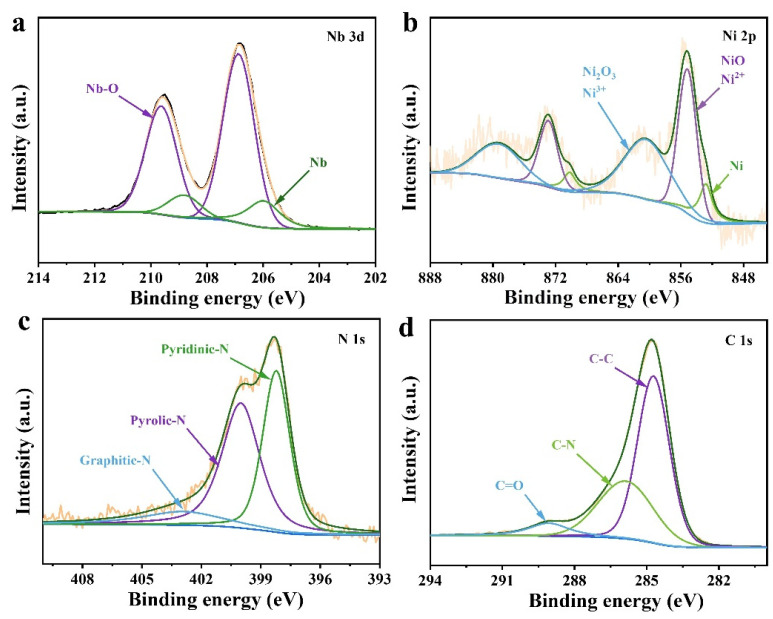
XPS spectra of Nb/Ni@NC nanofibers. High-resolution XPS spectra of: (**a**) Nb 3d; (**b**) Ni 2p; (**c**) N 1s; (**d**) C 1s.

To further explore the elemental composition and surface valence of the Nb/Ni@NC nanofibers, high-resolution X-ray photoelectron spectroscopy (XPS) was performed, as shown in Figure 4a–d. Figure 4a shows the high-resolution spectra of Nb 3d containing two pairs of characteristic peaks. The binding energies of 206 eV/208.8 eV and 206.9 eV/209.6 eV corresponded to the Nb-Nb and Nb-O bonds, respectively, and the existence of an Nb-O bond was attributed to the oxidation caused by the contact between the material surface and air. Figure 4b shows the high-resolution XPS spectra of Ni 2p containing three pairs of characteristic peaks. The binding energies of 855.2 eV/872.9 eV and 860.7 eV/879.6 eV were Ni^2+^ and Ni^3+^, respectively, while the binding energies of 852.9 eV/870.2 eV were elemental Ni; the formation of an Ni-O bond was also caused by the oxidation of the sample in air. The high-resolution image of N 1s in Figure 4c shows three separate characteristic peaks, indicating that there were three types of nitrogen atoms incorporated with binding energies of 398.2 eV, 400 eV, and 402.8 eV, which corresponded to pyridinic-N, pyrolic-N, and graphitic-N, respectively; these were all from polyacrylonitrile precursor. Figure 4d shows a high-resolution image of C1s containing three characteristic peaks at 284.8 eV, 285.9 eV, and 289 eV corresponding to the C-C, C-N, and C=O bonds, respectively. The XPS results again indicated the successful doping of two metallic elements as well as three forms of nitrogen in the Nb/Ni@NC nanofibers.

**Figure 5:**
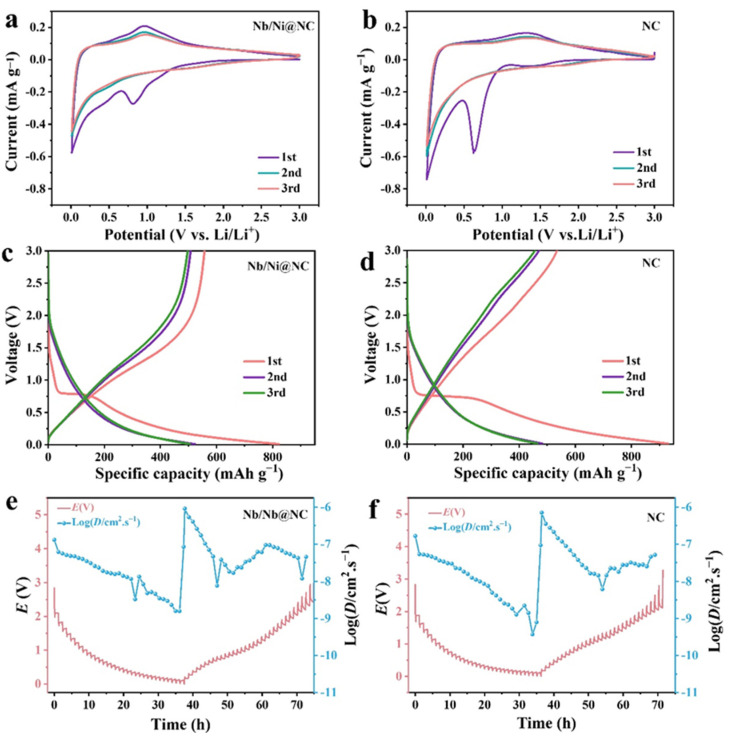
(**a**,**b**) Initial three CV curves of flexible Nb/Ni@NC and NC electrodes at 0.1 mV s^−1^; (**c**,**d**) initial three charge/discharge curves of flexible Nb/Ni@NC and NC electrodes at 0.2 mA g^−1^; (**e**,**f**) GITT curves and the corresponding Li^+^ diffusion coefficient of the flexible Nb/Ni@NC and NC electrode materials.

To study the electrochemical performance of the Nb/Ni@NC and NC electrodes, Li half-cells were assembled with the flexible Nb/Ni@NC and NC nanofiber membranes as self-supporting anodes, respectively, without usage of any conductive additives and binders. Figure 5a,b show the initial three cyclic voltammetry (CV) curves of the flexible Nb/Ni@NC and NC electrodes at a sweep speed of 0.1 mV s^−1^ within a voltage window of 0.01–3.0 V. The solid electrolyte interphase (SEI) in the Nb/Ni@NC and NC electrodes were obviously formed at 0.812 V and 0.634 V, respectively, and the peak current and peak area for the Nb/Ni@NC electrode were smaller than those of the NC, indicating that Nb/Ni@NC had a better reversibility and a higher initial coulombic efficiency. Figure 5c,d demonstrate the initial three charge/discharge profiles of the flexible Nb/Ni@NC and NC electrodes at 0.2 A g^−1^. Although the discharge plateau in the first cycle for the Nb/Ni@NC electrode was shorter than that of the NC electrode and the discharge capacity for the Nb/Ni@NC electrode was also lower than that of the control group, the discharge capacity in the subsequent cycles of the Nb/Ni@NC electrode was higher than that of the NC electrode, further indicating the higher initial coulombic efficiency of the Nb/Ni@NC electrode. In addition, electrochemical impedance spectroscopy (EIS) was used to analyze the electron/ion transport process in the Li half-cells. Generally, the resistance of the SEI film appeared in the semicircle of the high-frequency region while the charge transfer resistance was in the semicircle of the low-frequency region; the straight line of the low-frequency region was Warburg impedance [47]. As shown in Figure S5, both the SEI film resistance and charge transfer resistance of the Nb/Ni@NC electrode were smaller than those of the NC electrode. Moreover, constant current intermittent titration technology (GITT) was used on the two assembled cells to study whether the flexible Nb/Ni@NC electrode could accelerate the internal reaction in LIBs (Figure 5c,d). According to the Li^+^ diffusion coefficient formula: $\;D=\frac{4}{\pi \tau }{\left(\frac{m_{B}V_{m}}{M_{B}S}\right)}^{2}{\left(\frac{△E_{s}}{△E_{t}}\right)}^{2}$ (τ: the constant-current titration time, m_B_/M_B_: the mass and molar mass of the substance, S: the area of the electrode, and $△E_{s}$/$△E_{t}$: the steady-state voltage change and the voltage change during constant-current titration) [48], the diffusion coefficients for the Nb/Ni@NC and NC electrodes were calculated to be between 6.43 × 10^−6^~1.12 × 10^−8^ cm s^−1^ and 7.13 × 10^−7^~9.86 × 10^−11^ cm s^−1^, respectively. Compared with NC, the Nb/Ni@NC electrode had a higher ion diffusion coefficient, which was attributed to the introduction of Nb/Ni nanoparticles. The Nb and Ni metal nanoparticles could enhance the conductive property of the carbon nanofibers; the surface defects of the N-doped carbon conductive skeleton could provide more deposition sites for lithium; and the Nb, Ni, N could accelerate the charge transport together, thus promoting the transfer of Li^+^ and accelerating the electrochemical reaction kinetics in lithium-ion batteries.

**Figure 6:**
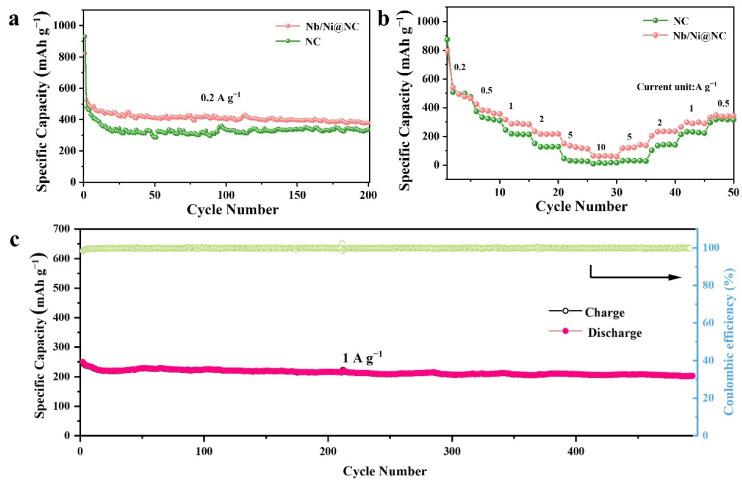
(**a**) Cycling performance of the flexible Nb/Ni@NC and NC electrodes at 0.2 A g^−1^; (**b**) rate capability of the flexible Nb/Ni@NC and NC electrodes; (**c**) cycling performance of the flexible Nb/Ni@NC electrode at 1.0 A g^−1^.

To further investigate the effects of Nb/Ni nanoparticle introduction on the electrochemical performance of the Nb/Ni@NC electrode, the cycling performance and rate capability at various currents for the flexible Nb/Ni@NC and NC electrodes were measured and compared. Figure 6a shows the cycle performance of the Nb/Ni@NC and NC electrodes at 0.2 A g^−1^. It can be seen that although the initial discharge capacity of the NC electrode was higher than that of the Nb/Ni@NC electrode (930.8 mAh g^−1^ vs. 822.41 mAh g^−1^), the reversible capacity for the NC electrode decayed faster. After 200 cycles, the Nb/Ni@NC electrode still had a reversible discharge capacity of 378.7 mAh g^−1^, which was higher than that of the NC electrode (336.8 mAh g^−1^). Moreover, the rate performances of the two cells were tested under different current densities (Figure 6b). When the current density was increased from 0.2 to 10.0 A g^−^^1^, the reversible discharge capacity for the Nb/Ni@NC electrode decreased from 884.7 mAh g^−1^ to 63.7 mAh g^−1^ and the capacity decay rate was lower than that of the NC electrode (from 874.1 mAh g^−1^ to 9 mAh g^−1^), showing a better rate performance by the Nb/Ni@NC electrode. Furthermore, when the current density was restored to 0.5 A g^−1^, the discharge capacity for the Nb/Ni@NC electrode was still higher than that of the NC electrode This was because the introduction of metal elements could not only enhance the conductivity of the nanofiber material, but also provided defects to modify the electronic structure; in addition, the composites played a key role in strengthening the affinity for lithium [49]. Therefore, the N-doped carbon supported with nanoparticle nanofibers could accelerate the transfer of charge and ions, thus improving the rate performance of the cell. In addition, the flexible Nb/Ni@NC electrode could also deliver a high reversible capacity and an excellent cycling stability under the long cycles at 1.0 A g^−1^ (Figure 6c). After 480 long cycles, the Nb/Ni@NC electrode still had a reversible capacity of 203.6 mAh g^−1^ with a capacity decay for each cycle of only 0.075%. In general, the flexible Nb/Ni@NC anode material had a better cycling stability and a better rate capability than the NC anode material; furthermore, the introduction of Nb and Ni nanoparticles played a crucial role in modifying the structure of the nanofiber, which allowed the flexible nanofiber membrane to exhibit an excellent electrochemical performance.

## 4. Conclusions

In this work, a flexible Nb/Ni@NC nanofiber membrane was successfully synthesized using electrospinning and thermal annealing and then used as a self-supporting anode material for lithium-ion batteries. The morphology of the nanofibers obtained by the electrospinning technology was uniform and controllable. The introduction of Nb and Ni nanoparticles could improve the electrical conductivity of the N-doped carbon nanofibers and modify the electronic structure by manufacturing carbon defects, resulting in the promotion of Li^+^ and electron transfer and enhancing the electrochemical kinetics. As a result, the flexible Nb/Ni@NC electrode had a higher initial coulomb efficiency, a better capacity retention and cycle stability, and a higher rate capability than the pure flexible NC electrode.

## Data Availability

Not applicable.

## Supplementary Materials

The following supporting information can be downloaded at: https://www.mdpi.com/article/10.3390/nano12213724/s1, Figure S1: Digital images of (**a**,**b**) self-supporting Nb/Ni@NC and NC nanofiber membranes as electrode materials; Figure S2: Folding tests of flexible Nb/Ni@NC and NC electrode materials; Figure S3: SEM images of NC nanofiber with low/high magnification; Figure S4: STEM image and EDS elemental mappings of Nb/Ni@NC nanofiber; Figure S5: Nyquist plots of the flexible Nb/Ni@NC and NC electrodes; Figure S6: Results of particle size analysis of Ni (**a**) and Nb (**b**) nanoparticles.
