# Supplementary material for: Nb and Ni Nanoparticles Anchored on N-Doped Carbon Nanofiber Membrane as Self-Supporting Anode for High-Rate Lithium-Ion Batteries
Source: Nanomaterials (Basel). 2022 Oct 23;12(21):3724. doi: 10.3390/nano12213724 (PMC9654905; doi:10.3390/nano12213724)
Supplement: Supplementary file 1 [file nanomaterials-12-03724-s001.zip › nanomaterials-1948474-supplementary.pdf]

Supplementary Materials

Nb and Ni Nanoparticles Anchored on N-Doped Carbon Nanofiber Membrane as Self-Supporting Anode for High-Rate Lithium-Ion Batteries

Yezheng Zhang, Shan Zhang, Shuo Zhao, Yingxue Cui, Jiabiao Lian and Guochun Li *

Institute for Energy Research, Jiangsu University, Zhenjiang 212013, China
* Correspondence: liguochun@ujs.edu.cn

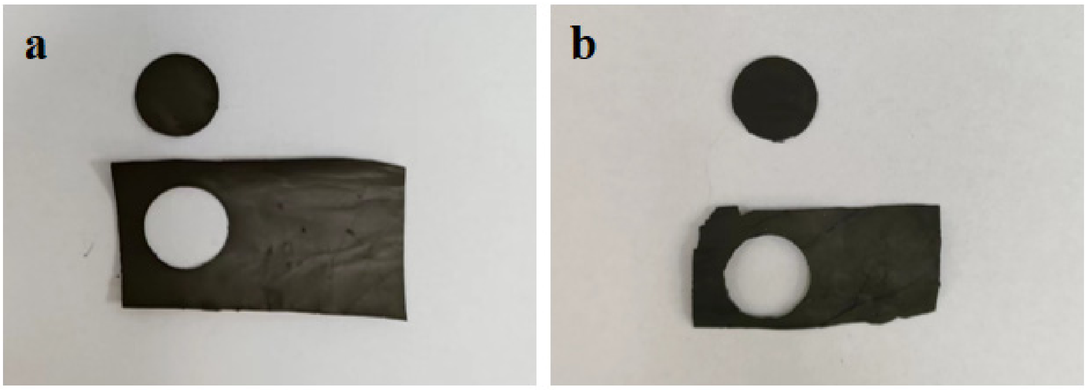

Figure S1. Digital images of (**a**,**b**) self-supporting Nb/Ni@NC and NC nanofiber membranes as electrode materials.

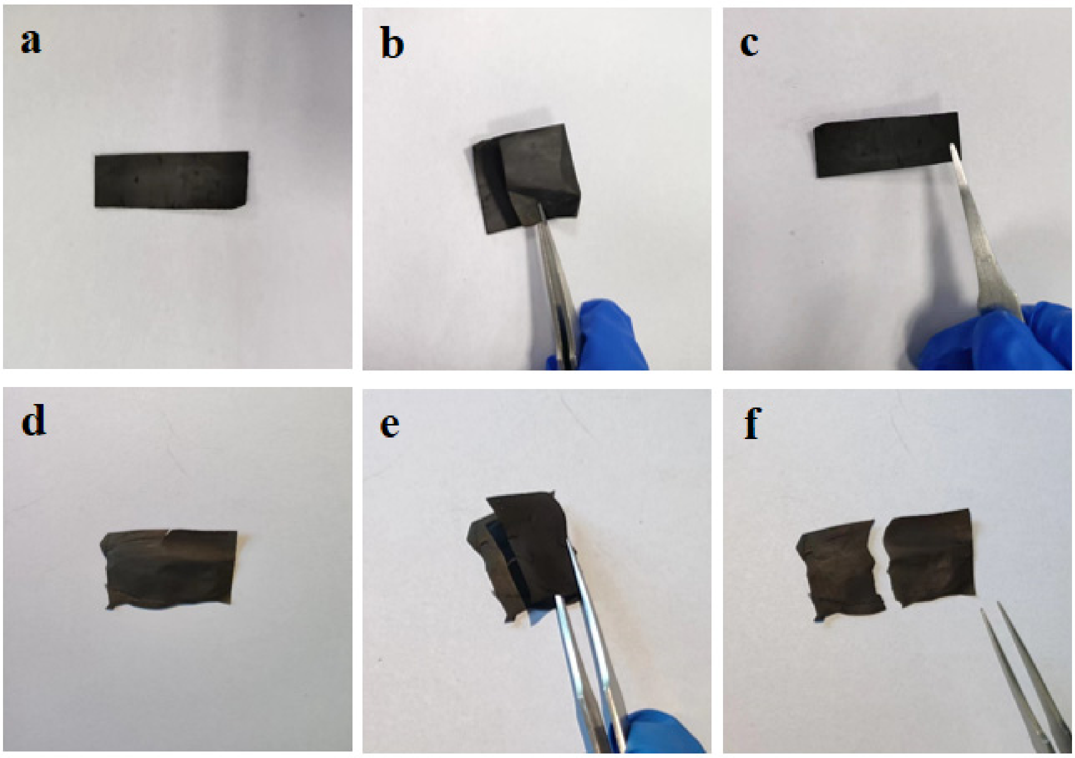

Figure S2. Folding tests of flexible Nb/Ni@NC and NC electrode materials.

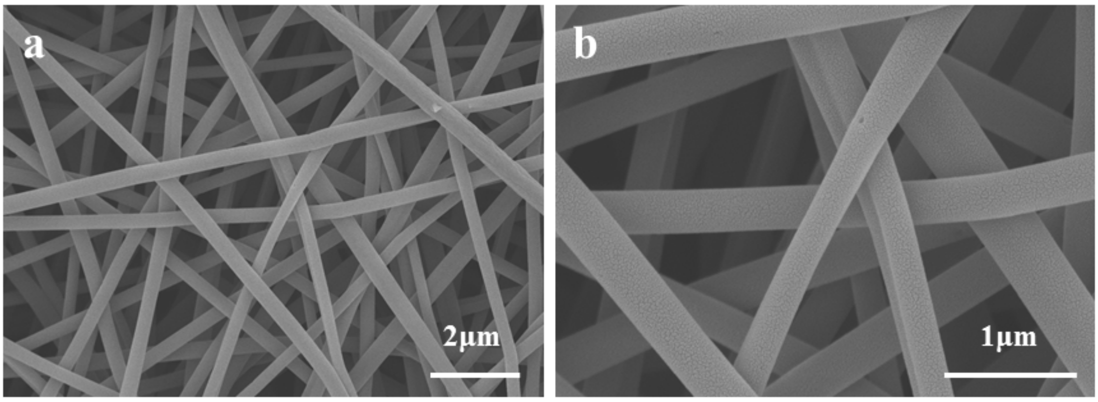

Figure S3. SEM images of NC nanofiber with low/high magnification.

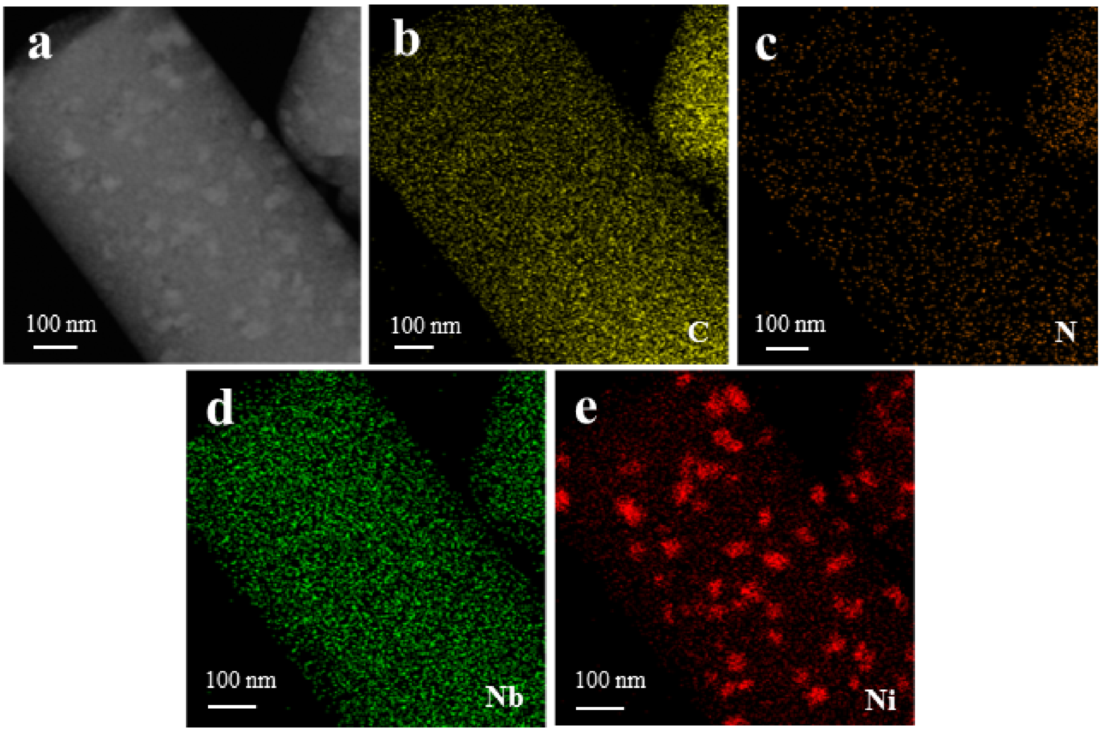

Figure S4. STEM image and EDS elemental mappings of Nb/Ni@NC nanofiber.

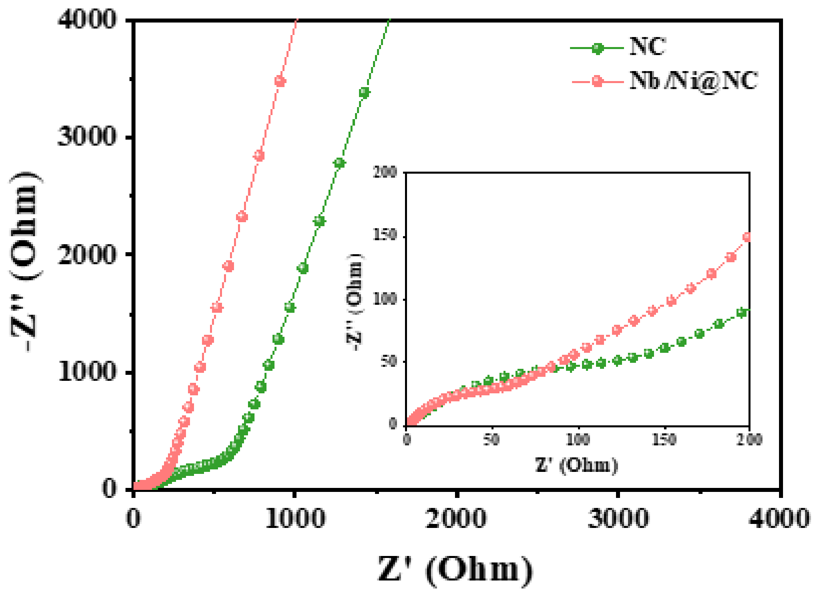

Figure S5. Nyquist plots of the flexible Nb/Ni@NC and NC electrodes.

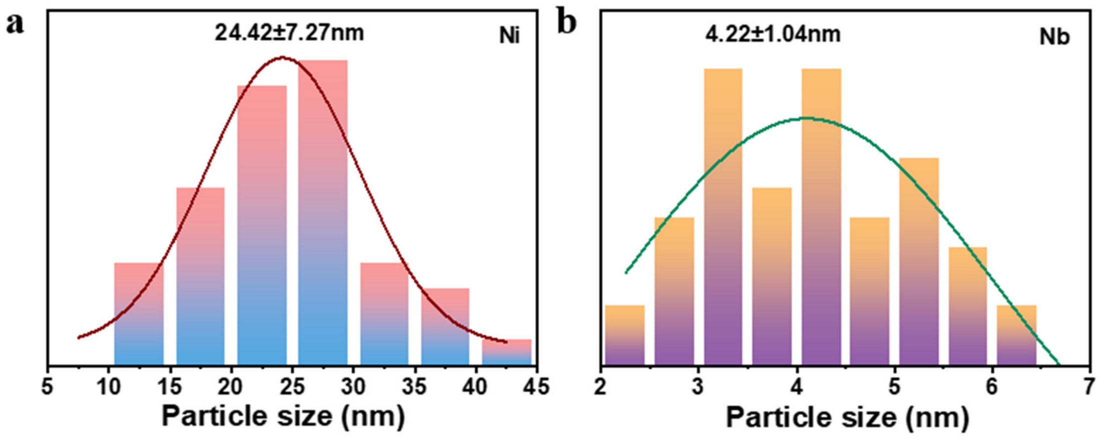

Figure S6. Results of particle size analysis of Ni (**a**) and Nb (**b**) nanoparticles.
